# *Panax notoginseng*: panoramagram of phytochemical and pharmacological properties, biosynthesis, and regulation and production of ginsenosides

**DOI:** 10.1093/hr/uhae170

**Published:** 2024-07-02

**Authors:** Guangfei Wei, Guozhuang Zhang, Mengzhi Li, Yuqing Zheng, Wenke Zheng, Bo Wang, Zhaoyu Zhang, Xiao Zhang, Ziying Huang, Tengyun Wei, Liping Shi, Shilin Chen, Linlin Dong

**Affiliations:** State Key Laboratory for Quality Ensurance and Sustainable Use of Dao-di Herbs, Key Laboratory of Beijing for Identification and Safety Evaluation of Chinese Medicine, Institute of Chinese Materia Medica, China Academy of Chinese Medical Sciences, No.16 Nanxiaojie, Dongzhimennei Ave., Beijing, 100700, China; State Key Laboratory for Quality Ensurance and Sustainable Use of Dao-di Herbs, Key Laboratory of Beijing for Identification and Safety Evaluation of Chinese Medicine, Institute of Chinese Materia Medica, China Academy of Chinese Medical Sciences, No.16 Nanxiaojie, Dongzhimennei Ave., Beijing, 100700, China; Nanyang Institute of Technology, Nanyang, No.80, Changjiang Road, Wulibao Street, Wancheng District, 473000, China; Zhangzhou Pien Tze Huang Pharmaceutical Co., Ltd, No. 1 Amber Road, Xiangcheng District, Zhangzhou, Fujian, 363099, China; Tianjin University of Traditional Chinese Medicine, No. 312, Anshan West Road, Nankai District, Tianjin, 301617, China; Hubei Institute for Drug Control, No.54, Dingziqiao Road, Zhongnan Road, Wuchang District, Wuhan, 430012, China; State Key Laboratory for Quality Ensurance and Sustainable Use of Dao-di Herbs, Key Laboratory of Beijing for Identification and Safety Evaluation of Chinese Medicine, Institute of Chinese Materia Medica, China Academy of Chinese Medical Sciences, No.16 Nanxiaojie, Dongzhimennei Ave., Beijing, 100700, China; Zhangzhou Pien Tze Huang Pharmaceutical Co., Ltd, No. 1 Amber Road, Xiangcheng District, Zhangzhou, Fujian, 363099, China; State Key Laboratory for Quality Ensurance and Sustainable Use of Dao-di Herbs, Key Laboratory of Beijing for Identification and Safety Evaluation of Chinese Medicine, Institute of Chinese Materia Medica, China Academy of Chinese Medical Sciences, No.16 Nanxiaojie, Dongzhimennei Ave., Beijing, 100700, China; Zhangzhou Pien Tze Huang Pharmaceutical Co., Ltd, No. 1 Amber Road, Xiangcheng District, Zhangzhou, Fujian, 363099, China; State Key Laboratory for Quality Ensurance and Sustainable Use of Dao-di Herbs, Key Laboratory of Beijing for Identification and Safety Evaluation of Chinese Medicine, Institute of Chinese Materia Medica, China Academy of Chinese Medical Sciences, No.16 Nanxiaojie, Dongzhimennei Ave., Beijing, 100700, China; State Key Laboratory for Quality Ensurance and Sustainable Use of Dao-di Herbs, Key Laboratory of Beijing for Identification and Safety Evaluation of Chinese Medicine, Institute of Chinese Materia Medica, China Academy of Chinese Medical Sciences, No.16 Nanxiaojie, Dongzhimennei Ave., Beijing, 100700, China; Institute of Herbgenomics, Chengdu University of Traditional Chinese Medicine, No. 37, 12 Qiao Road, Jinniu District, Chengdu, 611137, China; State Key Laboratory for Quality Ensurance and Sustainable Use of Dao-di Herbs, Key Laboratory of Beijing for Identification and Safety Evaluation of Chinese Medicine, Institute of Chinese Materia Medica, China Academy of Chinese Medical Sciences, No.16 Nanxiaojie, Dongzhimennei Ave., Beijing, 100700, China

## Abstract

*Panax notoginseng* is a famous perennial herb widely used as material for medicine and health-care food. Due to its various therapeutic effects, research work on *P. notoginseng* has rapidly increased in recent years, urging a comprehensive review of research progress on this important medicinal plant. Here, we summarize the latest studies on the representative bioactive constituents of *P. notoginseng* and their multiple pharmacological effects, like cardiovascular protection, anti-tumor, and immunomodulatory activities. More importantly, we emphasize the biosynthesis and regulation of ginsenosides, which are the main bioactive ingredients of *P. notoginseng*. Key enzymes and transcription factors (TFs) involved in the biosynthesis of ginsenosides are reviewed, including diverse CYP450s, UGTs, bHLH, and ERF TFs. We also construct a transcriptional regulatory network based on multi-omics data and predicted candidate TFs mediating the biosynthesis of ginsenosides. Finally, the current three major biotechnological approaches for ginsenoside production are highlighted. This review covers advances in the past decades, providing insights into quality evaluation and perspectives for the rational utilization and development of *P. notoginseng* resources. Modern omics technologies facilitate the exploration of the molecular mechanisms of ginsenoside biosynthesis, which is crucial to the breeding of novel *P. notoginseng* varieties. The identification of functional enzymes for biosynthesizing ginsenosides will lead to the formulation of potential strategies for the efficient and large-scale production of specific ginsenosides.

## Introduction

*Panax notoginseng* (Burk.) F.H. Chen, also known as Sanchi, is a perennial herb belonging to the Araliaceae family, extensively detailed in the classical Chinese pharmacopeia, Ben-Cao-Gang-Mu [1]. Originating from East Asia and North America around 25 million years ago, it predominantly thrives in China’s southwestern Yunnan and Guangxi regions, with the Yunnan variant highly regarded for its medicinal properties [2]. As both a medicinal and edible plant, *P. notoginseng* has garnered considerable interest for its applications in pharmaceuticals, functional foods, and health-care products [3]. Its notable effectiveness in enhancing blood circulation and alleviating pain renders it crucial in cardiovascular disease treatments, featuring prominently in traditional Chinese medicine prescriptions such as Yunnan Baiyao, Pientzehuang Compound, Danshen Dripping Pills, and Xuesaitong Injection [4]. Beyond its medical uses, *P. notoginseng* is prized for its health benefits and culinary applications. Its flowers are brewed into herbal teas that help cool the body and lower blood pressure, while its stems and leaves are utilized in cough-relieving teas. The roots, known for their anti-inflammatory and cardioprotective attributes, are often added to soups [5]. With its widespread popularity in various commercial sectors, including health and beauty, and dietary supplements, *P. notoginseng* enjoys broad recognition across Asia and in the West [6]. Responding to increasing demand, the industry has seen significant growth, with production reaching 28 000 tons and generating annual revenues of 16.2 billion Chinese yuan in 2017 [7].

The metabolites of *P. notoginseng* play a crucial role in its pharmacological efficacy. This plant harbors a diverse array of secondary metabolites, including ginsenosides, organic acids, esters, polysaccharides, amino acids, sterols, and flavonoids. These ingredients have significant therapeutic effects on the nervous system and immune response [8]. Ginsenosides stand out as the foremost bioactive elements, driving the medicinal effectiveness of this herb [9]. As the demand for *P. notoginseng* escalates, so does the interest in its agricultural production and the enhancement of biosynthetic methods for these active compounds. Such advancements are vital for the progression of herbal medicine and resource conservation. Plant metabolites are synthesized, transported, and metabolized through complex metabolic networks. Despite this, there remains a notable gap in the comprehensive analysis of the biosynthesis of these crucial active ingredients and their transcriptional regulation networks.

This review focuses on four principal topics: (i) bioactive constituents of *P. notoginseng*, (ii) its pharmacological benefits, (iii) the biosynthesis and regulation of ginsenosides, and (iv) biotechnological approaches to ginsenoside production. An in-depth exploration of the structures, distributions, biosynthetic routes, transcriptional regulatory frameworks, and biotechnological production techniques of ginsenosides is indispensable for the qualitative assessment and optimal exploitation of *P. notoginseng*.

## Bioactive ingredients

The therapeutic effects of *P. notoginseng* are mainly attributed to their bioactive components, such as saponins, amino acids, polysaccharides, and flavonoids [10]. Saponins are mainly divided into three dammarane-type tetracyclic triterpenoids, oleanane-type pentacyclic triterpenoids, and ocotilloltype pentacyclic triterpenoids, including ginsenosides Rb1, Rc, Rb2, Rg1, Re, Ro, and Rd, notoginsenoside R1, and majonoside R2. Amino acids include a non-protein amino acid (dencichine) and protein amino acids (such as arginine, aspartic acid, glutamic acid, and leucine). Polysaccharides include polysaccharide I, II (IIa, IIb), and III categories, and are mainly made up of glucose, galactose, and arabinose. The basic parent nucleus of flavonoids is 2-phenylketonuria and the basic skeleton of flavonoids is C6-C3-C6, and they mainly consist of quercetin, quercetin-7-glucoside, flavonoside quercetin-3-*O* sophoroside, and kaempferic acid. The types and contents of these bioactive components can be influenced by region, tissue, and development age, as discussed below.

### Saponins

**Figure 1:**
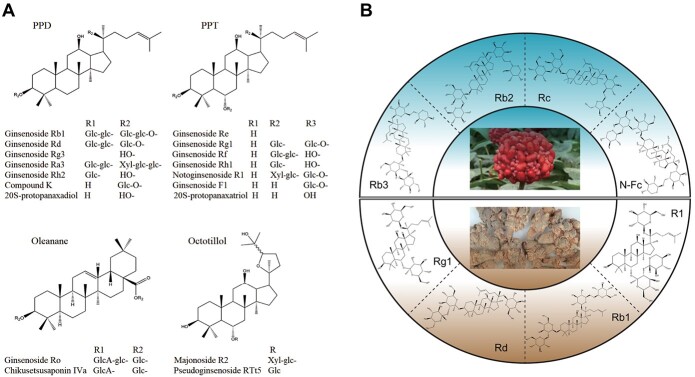
Specialized ginsenosides isolated from *P. notoginseng*. **A** Structures of major ginsenoside types. **B** Relative ginsenoside content distribution in the aerial and underground parts.

Saponins are the foremost active compounds in *P. notoginseng*, with >200 types identified by 2023 as noted in Supplementary Data Table S1 [4, 11–13]. These ginsenosides are classified into three main types: dammarane-type tetracyclic triterpenoids; oleanane-type pentacyclic triterpenoids; and ocotillol-type pentacyclic triterpenoids, as depicted in Fig. 1A [14]. Among them, dammarane-type ginsenosides are split into two subtypes: protopanaxadiol-type (PPD) and protopanaxatriol-type (PPT). PPD-type ginsenosides are typically bonded with sugar moieties at C-3, C-20, or both, whereas PPT-type ginsenosides feature sugars at C-3, C-6, and C-20. The ocotillol-type ginsenosides are distinguished by a five-membered epoxy ring on the C-20 side chain, and the oleanane-type by a modified C-20 side chain. Notably, PPT-type ginsenosides such as Rg1, R1, Re, and notoginsenoside R2 are found in concentrations ranging from 2.0 to 40.0 mg/g [15–17]. The most prevalent PPD-type ginsenosides, Rb1 and Rd, are found in concentrations of 26.7–30.6 and 5.7–8.4 mg/g, respectively [18]. Collectively, R1, Rb1, Rg1, Rd, and Re make up 90% of all ginsenosides in *P. notoginseng* [19].

The distribution and concentration of individual ginsenosides vary across different plant parts and developmental stages, as shown in Fig. 1B [20]. The total ginsenoside content in 3-year-old plants is 1.4 times higher than that in 2-year-old plants, with PPD-type ginsenosides predominantly distributed in aboveground parts and PPT-type ginsenosides mainly distributed in underground parts [21]. Leaves are particularly rich in PPD-type ginsenosides, including Rb3, Rc, and Rb2, while the main roots harbor significant levels of PPT-type ginsenosides such as Rg1, Re, and N-R1 [22, 23].

### Amino acids

Dencichine (β-*N*-oxalo-L-α-β-diaminopropionic acid, β-ODAP, C5H8N2O5), a non-protein amino acid with strong hemostatic activity and strong neuroexcitatory toxicity, is a characteristic component of *P. notoginseng* [24] (Supplementary Data Fig. S1 and Supplementary Data Table S1). The concentration of dencichine varies depending on the age and part of the plant, with the highest levels found in the flowers and leaves [24]. Specifically, dencichine content in 3-year-old flowers is 2.98%, which is 1.48 times greater than in 2-year-old flowers at 2.01%. Additionally, the method of drying significantly influences dencichine levels, the highest yield being observed with a drying treatment at 50°C and the lowest with freeze-drying at 40°C [25].

Furthermore, *P. notoginseng* is rich in protein amino acids, including arginine, aspartic acid, glutamic acid, and leucine (Supplementary Data Table S1). The concentration and composition of these amino acids vary across different plant parts and environments [26, 27]. Notably, the flowers possess the highest total amino acid content but lack arginine, whereas the roots are devoid of both arginine and methionine. To date, researchers have identified 19 amino acids in the roots of *P. notoginseng* [26].

### Polysaccharides

Polysaccharides are another crucial group of pharmacodynamically active components in *P. notoginseng*, as catalogued in Supplementary Data Table S1. Polysaccharides mainly include polysaccharide I, II (IIa, IIb), and III categories, and are predominantly made up of glucose, galactose, arabinose, lactose, rhamnose, xylose, and arabose, with the overall content of polysaccharides around 9.45% [28]. Notably, a starch-like polysaccharide (PNPN) along with six pectin variants, namely PNPA-1A, PNPA-1B, PNPA-2A, PNPA-2B, PNPA-3A, and PNPA-3B, have been identified [29]. Moreover, the crude polysaccharide extract of *P. notoginseng* consists of arabinose, galacturonic acid, d-mannose, d-glucose, and galactose, in a molar ratio of 2:1:2:83:7 [30]. As of 2022, researchers have documented a total of 41 polysaccharides from *P. notoginseng* [31, 32].

The polysaccharide levels in *P. notoginseng* are significantly affected by factors such as geographical location, time of harvest, and specific plant parts. The highest levels of polysaccharides are typically found in April and the lowest in July. Geographically, Yanshan shows the highest levels of polysaccharides, in contrast to the lower levels found in Guangxi [33]. Within the plant, the polysaccharide content varies, with ribs exhibiting the highest levels and stems and leaves the lowest [34].

### Flavonoids

The basic parent nucleus of flavonoids is 2-phenylketonuria and the basic skeleton of flavonoids is C6-C3-C6. A total of 13 varieties have been isolated from *P. notoginseng* [35, 36]. These primarily consist of quercetin, quercetin-7-glucoside, flavonoside quercetin-3-*O* sophoroside, kaempferic acid, quercetin-3-*O*-sophroside, kaempferol, kaempferol-7-*O*-α-l-rhamnoside, kaempferol-3-*O*-β-d-galactoside, kaempferol-3-*O*-β-d-galactose-(2 → 1)-glucoside, and quercetin-3-*O*-β-d-galactose-(2 → 1)-glucoside, as detailed in Supplementary Data Table S1. The concentration of these flavonoids varies by region, with Wenshan showing the highest levels at 0.7% and Honghe the lowest at 0.29%. The contents of flavonoids in different parts are significantly different: 1.77% in stem and leaf, 1.43% in flower, 0.50% in rhizome, 0.34% in fibril, and 0.19% in taproot [37].

## Pharmacological activities

**Figure 2:**
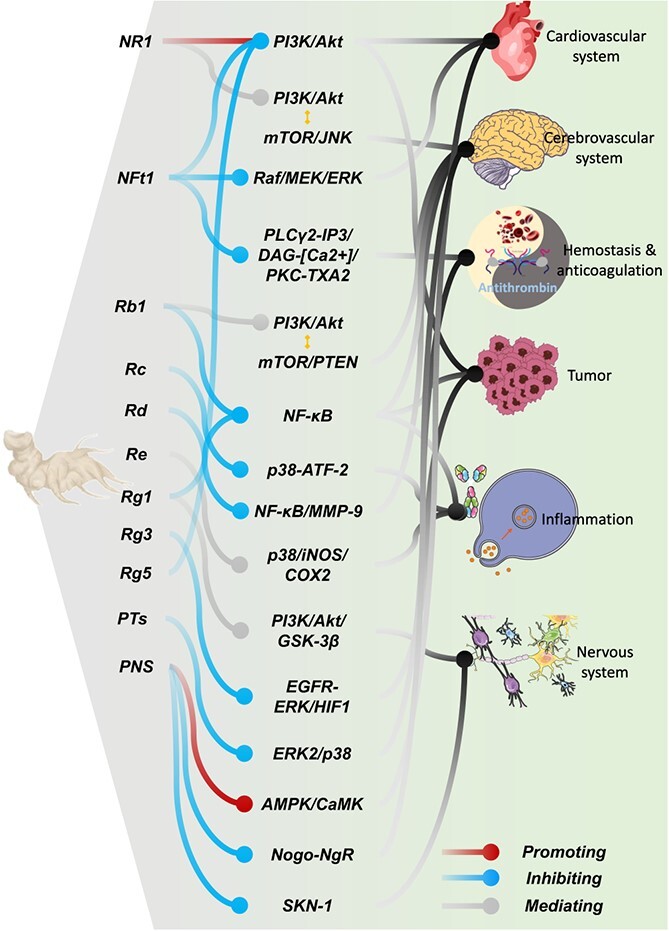
Summary of pharmacological effects and relevant mechanisms in *P. notoginseng*.

An increasing number of studies demonstrate that *P. notoginseng* has been widely used in the treatment of some chronic diseases, such as atherosclerosis, diabetes, cancer, and cardiovascular and cerebrovascular diseases [38]. Panax notoginseng saponins (PNS) are the major active ingredient of *P. notoginseng* and plays an important role in the medical and health fields. All kinds of PNS medicinal preparations, such as Sanqi-Tongshu capsules, Xuesaitong injection and Xuesaitong capsules, have been extensively used clinically. Modern research reveals that PNS possess a range of biological properties, including cardioprotective, cerebrovascular protective, neuroprotective, anti-tumor, anti-inflammatory, hemostatic, and anticoagulant effects. The following is an overview of these therapeutic effects and their relevant mechanisms (Table 1 and Fig. 2).

**Table 1:** Pharmacological activities of *P. notoginseng*.

**Effect type**	**Extract/compound**	**Material/model**	**Results**	**Mechanism**	**Reference**
Cardioprotective effect	Notoginsenoside R1	Mouse femoral artery endothelium denudation model	Inhibiting restenosis following percutaneous transluminal angioplasty	Hindering activation of the phosphatidylinositol 3-kinase (PI3K)/protein kinase B (Akt) signaling pathway	[39]
	Notoginsenoside Ft1	Human umbilical vein endothelial cells	Stimulating angiogenesis via HIF-1α-mediated VEGF expression	Inducing activation of the PI3K/AKT and Raf/MEK/ERK signaling pathways	[40]
	PNS	Left anterior descending ligation-operated mice	Enhancing endothelial cell migration and angiogenesis following myocardial infarction	Via phosphorylation of AMPK Thr 172 and CaMKII Thr 287	[41]
Cerebrovascular protective effect	PNS	Middle cerebral artery occlusion and oxygen–glucose deprivation/reperfusion model	Repairing the function of impaired nerves	Decreasing expression of Nogo-A and NgR	[42]
	PNS	Rat model of cerebral ischemia and SH-SY5Y cells	Providing neuroprotective effects	Inhibiting expression of NgR1, RhoA, and ROCK2	[43]
	Notoginsenoside R1	Hypoxic–ischemic brain damage in neonates	Inhibiting neuronal apoptosis and promoting cell survival	Via the PI3K-Akt–mTOR/JNK signaling pathways by targeting estrogen receptors	[44]
	Ginsenoside Rd	Sprague–Dawley rats with focal cerebral ischemic injury	Attenuating the pathogenesis of cerebral ischemia-induced blood–brain barrier damage	Inhibiting proteasome activity and suppressing the NF-κB/MMP-9 pathway	[45]
	Ginsenoside Rb1	Rats with induced photothrombotic stroke	Alleviating the morphological lesion and cognitive and sensorimotor deficits	Via the modulation of the Akt/mTOR/PTEN signaling pathway	[46]
	Ginsenoside Rg1	Cerebral ischemia/reperfusion injury model	Preventing IR-induced neurological injuries	Blocking NF-κB nuclear translocation and phosphorylation of IκBα	[47]
Neuroprotective effect	PNS	β-Mediated neurotoxicity in *Caenorhabditis elegans*	Inhibiting Alzheimer’s disease-like symptoms	Involving the SKN-1 signaling pathway and expression of Alzheimer’s disease-related circRNAs	[48]
	Ginsenosides Rg1 and Rg2	Rats with Alzheimer’s disease	Enhancing cognitive function and reducing hippocampal amyloid-β deposition	Via modulation of associated metabolic pathways	[49]
	Ginsenoside Rg1	Rats exposed to isoflurane anesthesia	Combating cognitive decline	Via antioxidant, anti-inflammatory and anti-apoptotic effects, mediated by the PI3K/AKT/GSK-3β pathway	[50]
Anti-tumor effect	PNS	Mice with AOM/DSS-induced colorectal cancer	Reducing the immunosuppress	Inhibiting the expression of IDO1	[51]
	Ginsenoside Rb1	H9c2 and liver carcinoma HepG-2 cells	Curtailing production of tumor necrosis factor-*α* induced MMP-9	Suppressing the RNA-dependent protein kinase and NF-κB signaling pathways	[52]
	Notoginsenoside R1	Hepatocellular carcinoma cells	Boosting antihepatoma efficacy	Inactivation of the PI3K/Akt pathway by suppressing miR-21	[53]
	Ginsenoside Rg3	Hepatocellular carcinoma cells	Decreasing NHE1 expression	Blocking the epidermal growth factor receptor pathway, including phosphorylated extracellular signal-regulated kinase and hypoxia-inducible factor 1 alpha	[54]
	Ginsenoside Rg5	Breast cancer in a mouse model	Inducing apoptosis and autophagy	Disrupting the PI3K/Akt signaling pathway	[55]
Anti-inflammatory effect	Ginsenoside Re	BV2 microglial cells	Neuroprotective events in neuroinflammation occurred	Via the phospho-p38, iNOS, and COX2 signaling pathways	[56]
	Ginsenoside Rg1	Rodent model mimicking alcoholic hepatic injury	Alleviating symptoms of alcoholic hepatitis and TNBS-induced colitis	Inhibiting the NF-κB pathway	[57]

**Table 1:** Continued.

**Effect type**	**Extract/compound**	**Material/model**	**Results**	**Mechanism**	**Reference**
	Ginsenoside Rc	HEK293 cells transfected with various inducers of inflammation	Exerting anti-inflammatory actions	Suppressing TANK-binding kinase 1/IκB kinase ε/interferon regulatory factor-3 and p38/ATF-2 signaling pathway	[58]
Hemostasis and anticoagulation	Panaxatriol saponins (Rg1, Re, and R1)	Rabbit or human platelets	Inhibiting platelet aggregation	Reducing intracellular calcium mobilization and deactivating the ERK2/p38 pathway	[59]
	Notoginsenoside Ft1	Rats	Influencing coagulation processes	Enhancing the PLCγ2-IP3/DAG-[Ca2+]/PKC-TXA2 signaling pathway	[60, 61]

### Cardioprotective effect

Cardiovascular diseases are the primary causes of mortality in developed nations, accounting for half of all deaths [62]. Research has demonstrated that *P. notoginseng* has potent protective effects on myocardial cells and combats cardiovascular ailments through a variety of complex signaling pathways. For instance, notoginsenoside R1 is acknowledged as a promising therapeutic to inhibit restenosis following percutaneous transluminal angioplasty, by hindering the activation of the phosphatidylinositol 3-kinase (PI3K)/protein kinase B (Akt) signaling pathway [39]. Moreover, notoginsenoside Ft1 boosts the expression of vascular endothelial growth factor (VEGF), stimulated by HIF-1α, linked to the PI3K/Akt and Raf/MEK/ERK signaling pathways [40]. Additionally, PNS and its main constituents, ginsenosides Rg1 and R1, are known to significantly enhance endothelial cell migration and angiogenesis following myocardial infarction through the phosphorylation of AMPK Thr 172 and CaMKII Thr 287 [41].

### Cerebrovascular protective effect

Cerebrovascular diseases rank as significant causes of disability and the third leading cause of mortality in developed nations [63]. PNS have shown efficacy in mitigating neurological deficits, cerebral infarctions, edema, and neuronal death [42, 43]. For instance, ginsenoside Rd is noted for potentially reducing tau phosphorylation induced by cerebral ischemia through the PI3K/Akt/GSK-3β pathway, and for lessening damage to the blood–brain barrier via the NF-κB/MMP-9 pathway [44, 45]. Additionally, ginsenoside Rb1 influences the Akt/mTOR/PTEN signaling pathway, offering therapeutic benefits for various neurological conditions [46]. Ginsenoside Rg1 also plays a role in preventing IR-induced neurological injuries by blocking NF-κB nuclear translocation and the phosphorylation of IκBα, and by lowering glutamate and aspartate levels [47].

### Neuroprotective effect

The incidence of neurodegenerative disorders such as Alzheimer’s and Parkinson’s is on the rise in developed nations, a trend attributed to longer life expectancies [64, 65]. Research into the therapeutic benefits of ginsenosides for these conditions has identified potential mechanisms involving the SKN-1 signaling pathway and the expression of Alzheimer’s disease-related circRNAs [48]. Ginsenosides Rg1 and Rg2 are noted for enhancing cognitive function and reducing hippocampal amyloid-β (Aβ) deposition through modulation of associated metabolic pathways [49]. Additionally, ginsenoside Rg1 combats cognitive decline through its antioxidant and anti-inflammatory actions mediated by the PI3K/Akt/GSK-3β pathway [50]. Ginsenoside Rb1 is also used in managing Huntington’s disease, traumatic brain injuries, and ischemia [66], while ginsenoside Rd is known to mitigate excitatory toxicity, modulate nerve growth factors, and promote nerve regeneration [67].

### Anti-tumor effect

Research has demonstrated that *P. notoginseng* has significant anti-tumor effects against several types of cancer, including liver cancer [8]. PNS influence immune responses and curb tumor proliferation through various mechanisms. For example, they reduce the immunosuppression of Treg cells within the colorectal cancer environment by inhibiting the expression of IDO1, which is controlled by signal transducer and activator of transcription 1 (STAT1) [51]. Rb1 curtails the production of tumor necrosis factor-α (TNF-α)-induced MMP-9 by modulating the RNA-dependent protein kinase and the NF-κB signaling pathways [52]. R1 boosts antihepatoma efficacy by activating the PI3K/Akt pathway and suppressing miR-21, clarifying its role in combating hepatocellular carcinoma [53]. Rg3 decreases NHE1 expression by blocking the epidermal growth factor receptor pathway, including phosphorylated extracellular signal-regulated kinase and hypoxia-inducible factor 1α in hepatocellular carcinoma [54]. Additionally, ginsenoside Rg5 promotes apoptosis and autophagy by disrupting the PI3K/Akt signaling pathway [55].

### Anti-inflammatory effect

Inflammation plays a crucial role in the body’s immune response and is linked to various health conditions. Ginsenosides are effective in reducing pro-inflammatory cytokines, such as IL-1β and TNF-α, thus mitigating cerebral ischemic injuries in the brain [68, 69]. Rb1, along with its metabolite compound K, blocks the activation of NF-κB, MAP kinases (ERK, JNK, and p-38), IKK-β, and interleukin-1 receptor-associated kinase-1 in lipopolysaccharide (LPS)-stimulated murine peritoneal macrophages [70]. The anti-inflammatory properties of ginsenoside Rd stem from its ability to downregulate NF-κB, leading to decreased expression of iNOS and COX-2 [71]. Ginsenoside Re is neuroprotective against 1 μg/ml LPS-treated microglial cells, and the neuroprotective events in neuroinflammation occurred via the phospho-p38, iNOS, and COX2 signaling pathways [56]. Furthermore, ginsenoside Rg1 has been shown to alleviate symptoms of alcoholic hepatitis and TNBS-induced colitis by inhibiting the NF-κB pathway [57]. Additionally, ginsenoside Rc targets the TANK-binding kinase 1/IκB kinase ε/interferon regulatory factor-3 and p38/ATF-2 signaling pathways, exerting its anti-inflammatory effects [58].

### Hemostasis and anticoagulation

Atherosclerosis, a major contributor to cardiovascular and cerebrovascular diseases, can be mitigated by *P. notoginseng* [72]. Research demonstrated that panaxatriol saponins (Rg1, Re, and R1) effectively inhibit platelet aggregation by reducing intracellular calcium mobilization and deactivating the ERK2/p38 pathway [59]. Further studies indicate that Rg1 and Rg2 significantly extend blood clotting times, with Rg2 exhibiting a more potent anticoagulant effect than Rg1 [73]. Additionally, Rg2, Rg3, and protopanaxatriol are identified as potential natural inhibitors of clotting, contributing to their anti-platelet aggregation properties [74]. Rh1 and ginsenoside F1 also show capabilities in preventing platelet aggregation [75]. Moreover, notoginsenoside Ft1 influences coagulation processes by enhancing the PLCγ2-IP3/DAG-[Ca^2+^]/PKC-TXA2 signaling pathway [60, 61].

## Biosynthesis and regulation of saponins

The most important active ingredients of *P. notoginseng* that exert pharmacological effects are ginsenosides, a series of triterpenoid saponins. However, the resources of *P. notoginseng* are currently limited. In order to better solve the source problem of ginsenosides, we need to comprehend the biosynthesis and regulation of ginsenosides in *P. notoginseng*, providing more clues for the breeding of novel *P. notoginseng* varieties with high ginsenoside contents and potential biotechnological methods for efficient and large-scale production of ginsenosides. The genes and transcription factors (TFs) involved in the ginsenoside biosynthesis pathways of *P. notoginseng* primarily govern the enzymes that facilitate the structurally diverse biosynthesis of ginsenosides. Additionally, environmental factors significantly influence ginsenoside production through interactions within hormonal signal-transcription regulatory networks. The biosynthesis and regulation of ginsenosides of *P. notoginseng* are reviewed below.

### Biosynthesis of ginsenosides

**Figure 3:**
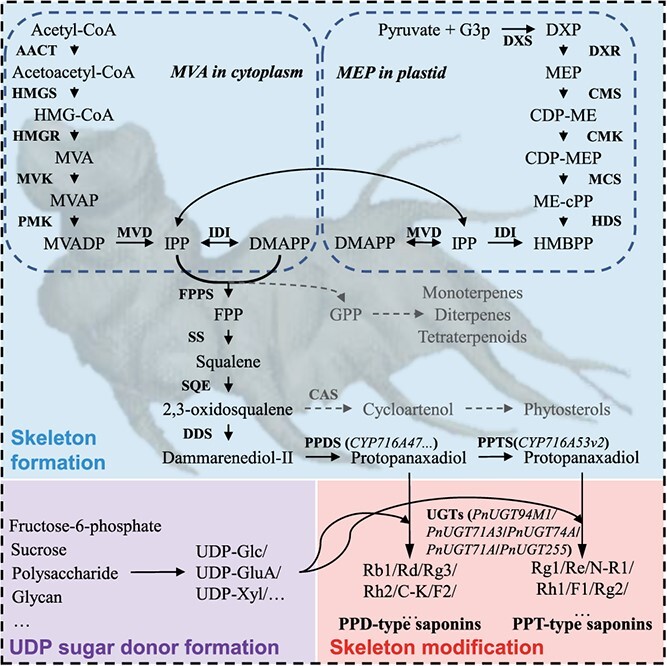
Possible biosynthesis pathway of ginsenosides in *P. notoginseng*.

Ginsenosides in the *Panax* genus and their biosynthesis have been reported previously [76]. The biosynthesis of ginsenosides primarily encompasses three stages: formation of the ginsenoside skeleton, glycosome synthesis, and modification of the skeleton [76]. The synthesis of ginsenosides predominantly occurs through the mevalonic acid (MVA) pathway in the cytoplasm and the methylerythritol phosphate (MEP) pathway in plastids, as illustrated in Fig. 3.

#### Skeleton formation

The biosynthesis of ginsenosides involves upstream MVA and MEP pathways, which are basically similar in *Panax* [77]. Initially, the terpenoid precursors isopentenyl diphosphate (IPP) and its isomer dimethylallyl diphosphate (DMAPP) are synthesized. These are then converted into geranyl pyrophosphate and farnesyl pyrophosphate (*FPP*). Two molecules of *FPP* are combined by squalene synthase (*SS*) to form squalene [78]. This squalene is transformed into 2,3-oxidosqualene by squalene epoxidase (*SE*) and dammarenediol synthase (*DS*). Finally, 2,3-oxidosqualene undergoes cyclization, hydroxylation, and glycosylation to become ginsenosides [79].

3-Hydroxy-3-methylglutaryl CoA reductase (*HMGR*) is a crucial rate-limiting enzyme in ginsenoside biosynthesis [80]. The enhancement of *PnHMGR* expression leads to an increase in ginsenoside production in *P. notoginseng* [81]. Key enzymes such as *PnFPS*, *PnSS*, *PnSE1*, *PnSE2*, and *PnDS* are involved in this process and exhibit organ-specific expression patterns, showing particularly high levels in the flowers of 4-year-old *P. notoginseng* [82]. *PnSE1* is present in all organs but predominantly in flowers, while *PnSE2* shows relatively low expression [83]. The overall ginsenoside content is largely dependent on the activity of farnesyl diphosphate synthase (*FPS*), followed by *SS* and *DS*, with the concentration of Rb1 specifically linked to the activities of *HMGR* and *FPS* [84]. Research has demonstrated that the genes *PnFPS*, *PnSS*, *PnSE1*, *PnSE2*, and *PnDS* have tissue-specific expression patterns and show significantly higher levels in the flowers and leaves compared with the roots and fibrils, being 5.2 times greater [21, 85].

#### Cytochrome P450

After the initial formation of the basic ginsenoside skeleton, it is further transformed into ginsenosides with great diversity in structure and function in the *Panax* genus via hydroxylation by cytochrome P450 enzymes (CYPs) and glycosylation by uridine diphosphate (UDP)-dependent glycosyltransferases (UGTs). CYP enzymes are part of large and functionally diverse gene families [79]. The subfamily CYP716, belonging to the CYP85 clade, is a major contributor to the diversification of triterpenoid biosynthesis [86]. Specifically, three CYP716s – CYP716A47 (protopanaxadiol synthase, PPDS), CYP716A53v2 (protopanaxatriol synthase, PPTS), and CYP716A52v2 (oleanolic acid synthase, OAS) – have been identified [87–89]. Dammarenediol-II undergoes hydroxylation by CYP716A47 and CYP716A53v2 to form protopanaxadiol and protopanaxatriol in four common *Panax* species (*P. notoginseng*, *P. ginseng*, *P. quinquefolius*, and *P. japonicus*) [90]. Distinct from CYP716A47 and CYP716A53v2, CYP716A52v2 serves as a multifunctional oxygenase involved in the biosynthesis of oleanolic acid in *P. ginseng*, *P. quinquefolius*, and *P. japonicus*, but is not expressed in *P. notoginseng*. So CYP716 plays a pivotal role in the diversification of ginsenosides in *Panax* species. Thus, the expression of some differential genes in downstream synthetic pathways in the *Panax* genus will result in the unique monomer ginsenosides of *Panax* species.

The CYP716 subgroup is not only hydroxylated at different carbon sites in different members of the *Panax* genus, but also has different expression levels in different plant tissues (leaf and root tissues). Thus, dammarane-type [synthesized by Y subgroup (CYP716A47) and S subgroup (CYP716A53v2)] and oleanane-type [synthesized by A subgroup (CYP716A52v2)] ginsenosides presented different concentrations in leaf and root tissues [91]. The CYP716 genes, including *PN006374*, *PN008424*, *PN011429*, and *PN029913*, show high expression levels in the roots and are likely crucial for the biosynthesis of PPT-type ginsenosides [92, 93].

**Figure 4:**
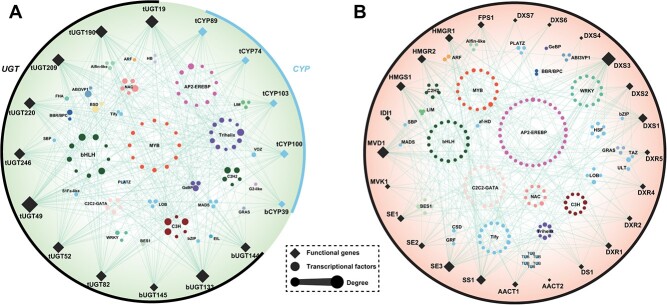
Regulation relationship between TFs and genes involved in ginsenoside synthesis from *P. notoginseng*. **A** Regulatory network of CYPs and UGTs with TFs. **B** Regulatory network of genes from ginsenoside skeleton formation and TFs. A total of 103 TFs were identified, the transcript abundances of which were significantly correlated with functional genes (Pearson’s |*r*| > 0.8, adjusted *P* < 0.05). Edges represent significant correlations, and node size is mapped to degree.

### UDP-dependent glycosyltransferases

The UGTs are instrumental in forming various monomeric ginsenosides by attaching monosaccharides to aglycones, primarily at position C-3 or C-20 for PPD-type ginsenosides, and at C-6 and/or C-20 for PPT-type ginsenosides [94]. Propanaxanediol and propanaxatriol are glycosylated by various UGT enzymes to form PPD-type and PPT-type ginsenosides, which exist universally in *Panax* plants. For example PnUGT71A3 is capable of catalyzing both the C6 hydroxyl glycosylation of PPT and F1 into Rh1 and Rg1, and the C20 hydroxyl glycosylation of Rg3 into Rd [95]. PnUGT94M1 facilitates the C2 hydroxyl rhamnosylation of Rg1 and Rh1 into Re and Rg2, respectively. *PnUGT74A* is able to convert CK into F2, PnUGT94C transforms Rh2 into Rg3 and F2 into Rd, and *PnUGT71A* modifies PPT into F1 and PPD into CK [96]. The presence of *PnUGT255* is associated with R1 levels, and PnUGT190 significantly influences Rd concentrations [97]. Elevated expression of three *UGT71* genes (*PN000453*, *PN025151*, and *PN025152*) and three *UGT74* genes (*PN000316*, *PN024572*, and *PN033259*) in the aerial parts is linked to the biosynthesis of PPD-type ginsenosides [98].

### Transcriptional regulation

The biosynthesis and accumulation of ginsenosides are characterized by distinct spatio-temporal patterns. The expression of genes involved in biosynthesis is regulated by a variety of TFs and non-coding RNAs (ncRNAs) through intricate regulatory networks. These TFs and ncRNAs can respond to both biotic and abiotic signals, playing crucial roles in the regulation of ginsenoside production.

#### Transcription factors

TFs regulate gene transcription through complex networks, thereby influencing ginsenoside concentrations. Key TF families, such as WRKY, basic helix–loop–helix (bHLH), myeloblastosis (MYB), and AP2/ERF (ERF1), are crucial in ginsenoside biosynthesis. A comprehensive identification has revealed 2150 TFs across 57 families in *P. notoginseng* [99].

Overexpression of *PnMYB1* in *P. notoginseng* upregulates the expression of *PnFPS*, *PnSS*, and *PnDS* genes and subsequently increases the levels of ginsenosides R1, Rg1, Re, Rb1, and Rd [100]. *PnMYB2* enhances the expression of *PnSS* and *PnSE1* by interacting with their promoters [101]. Conversely, inhibition of *PnMYB4* can increase expression of ginsenoside biosynthetic genes, including *PnSS*, *PnSE*, and *PnDS*, and thus boosts ginsenoside content. Both *PnMYB4* and *PnMYB1* collaborate with *PnbHLH* to fine-tune ginsenoside biosynthesis [102]. In transgenic cell lines expressing *PnbHLH1*, the transcription of key biosynthetic genes – *PnDS*, *PnSS*, *PnSE*, and *PnFPS* – is elevated, doubling the total ginsenoside content compared with wild-type cell lines [103]. The PnbHLH TF indirectly augments ginsenoside levels by regulating key biosynthetic genes at multiple points [104]. With *PnWRKY1* overexpression in *P. notoginseng*, the expression of *PnDS*, *PnSS*, and *PnSE* escalates by 3.1, 4.0, and 4.5 times, respectively, influencing ginsenoside concentrations [105]. In cell lines overexpressing *PnERF1*, transcription levels of *PnDS* and *PnSS* are increased by 1.6 and 1.9 times, respectively, enhancing the content of six ginsenosides: Rg3, Rh1, Rd, Rg1, F1, and Re [106]. Additionally, *PnERF2* and *PnERF3* show significant correlation with *DS* and *SE* expression in various tissues of methyl jasmonate (MeJA)-induced *P. notoginseng* [107].

Data from the *Panax* genome and transcriptome database (NCBI accession number PRJNA488357) significantly enhance gene research, although reports on the transcriptional regulatory networks for ginsenoside biosynthesis are scarce. Utilizing multi-omics data and patterns of ginsenoside accumulation, co-expression networks within *P. notoginseng* have been developed to hypothesize the regulatory interactions between TFs and genes involved in ginsenoside biosynthesis (Fig. 4 and Supplementary Data File 2). TFs were categorized using the Plant Transcription Factor Database PlantTFDB (http://planttfdb.gao-lab.org/), and Pearson’s correlation coefficients were calculated to determine the relationships between TFs and ginsenoside biosynthetic genes using tools from OmicStudio (https://www.omicstudio.cn/tool) [108]. Analysis of the *P. notoginseng* transcriptome database identified 103 TFs that are predicted to have significant positive or negative correlations with the regulation of ginsenoside biosynthetic genes (Pearson’s |*r*| > 0.8, adjusted *P* < 0.05). This correlation underscores the complexity of the transcriptional regulatory network that mediates ginsenoside biosynthesis. Each biosynthetic gene is influenced by multiple TFs, which can either enhance or suppress its activity. Further research is necessary to understand the regulatory functions and interactions among TFs in *P. notoginseng* to clarify how they control the synthesis of specialized ginsenosides. Additionally, many TFs are capable of simultaneously regulating multiple biosynthetic genes; thus, uncovering the molecular mechanisms that influence these transcriptional regulations is crucial for developing innovative breeding strategies.

#### Non-coding RNAs

In the roots of *P. notoginseng*, miR156 and miR166 are recognized as the predominant miRNA families, with miR156i and miR156g being the most abundant within these groups [109]. The miR156 family, alongside one of its target genes from the squamosa promoter-binding protein-like (SPL) category, exhibits inversely related expression levels that are closely associated with the increase in root biomass content [110].

### Environmental regulation

**Figure 5:**
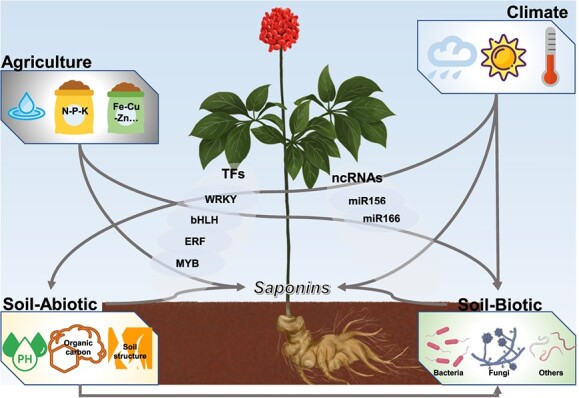
Environmental regulation (climatic factors, edaphic factors, and agricultural practice) of ginsenoside biosynthesis.

Environmental factors collaboratively influence the synthesis of active ingredients through hormone signal-transcription regulatory networks in medicinal plants [111]. This environmental regulation encompasses responses to heavy metals, various environmental conditions, and fertilizer applications, as illustrated in Fig. 5.

#### Heavy metal regulation

The relationship between heavy metals and plant growth, alongside their impact on metabolite synthesis, can be described as ‘low promotion and high inhibition’ [112]. At low concentrations, cadmium (Cd) increases the expression of *DS*, whereas high concentrations inhibit it; additionally, the presence of Cd significantly reduces the expression of cytochrome P450 enzymes [113]. However, the expression levels of *DS* and P450 genes do not show a strong correlation with the contents of R1, Rb1, Rg1, and total ginsenoside (*P* > 0.5). Endogenous nitric oxide (NO) boosts the accumulation of Rb1 but lowers the levels of Re and Rg1. Furthermore, endogenous NO increases the transcription of β-amyrin synthase (*β-AS*), cycloartenol synthase (*CAS*), and squalene epoxidase (*SE*), yet decreases the transcription of *DS* under Cd stress [114].

#### Environmental factor regulation

Recent studies have demonstrated that environmental factors such as light, temperature, water, and salinity influence the synthesis and accumulation of secondary metabolites in medicinal plants [115]. Both the rhizosphere and endophytic microflora of *P. notoginseng* play a role in enhancing plant health, biomass production, and the synthesis or biotransformation of ginsenosides, either directly or indirectly [116]. Climate, soil, and microbial interactions synergistically affect the contents of ginsenosides in *P. notoginseng* [117]. The concentrations of total and individual ginsenosides in the taproot of *P. notoginseng* show regional variations, with higher ginsenoside levels achievable by increasing temperature in January, atmospheric humidity, and soil calcium content, and by reducing latitude and average July temperatures [118]. Additionally, sunshine duration is a significant factor in ginsenoside production, with content increasing alongside longer exposure to sunlight [119].

#### Fertilizer regulation

Optimal fertilizer application during the cultivation of *P. notoginseng* enhances both biomass and ginsenoside content [120]. The expression of *PnbZIP* TFs in roots varies under different nitrogen fertilizer conditions; notably, *PnbZIP46* is significantly upregulated under ammonium nitrogen fertilizer stress, which may contribute to nitrogen stress resistance [121]. Adjusting the nitrogen/potassium application ratio to 1:2 can improve the synergistic effect on both biomass and ginsenoside content in *P. notoginseng* [122]. A lower nitrogen/potassium ratio enhances photosynthesis, sugar accumulation, and the expression of genes involved in ginsenoside biosynthesis. Additionally, the use of ammonium and nitrate fertilizers stimulates the tricarboxylic acid (TCA) cycle, thereby increasing both biomass and ginsenoside content in *P. notoginseng* roots [123]. Conversely, high nitrogen concentrations can inhibit biomass and ginsenoside content; excessive nitrogen reduces root biomass and ginsenoside accumulation, likely due to decreased nitrogen efficiency and reduced photosynthetic capacity [124].

**Table 2:** Biosynthesis of ginsenosides by metabolic engineering

**Types**	**Cultured material**	**Operational method**	**Products**	**Reference**
Plants	Suspension cultures	2-Hydroxyethyl jasmonate elicitation	Increasing P6H and Rb1 and Rg content	[125]
	Suspension cultures	2-Hydroxyethyl jasmonate elicitation	The total ginsenoside content and Rb/Rg ratio increased about 60 and 30%	[126]
	Suspension cultures	Phenobarbital treatment	Enhancing the production of protopanaxatriol-type (Rg1 + Re) ginsenosides	[127]
	Suspension cultures	MeJA treatment	About 9-fold PPD-type and 2-fold PPT-type ginsenosides	[128]
	Suspension cultures	2-Hydroxyethyl jasmonate and jasmonic acid treatment	Enhancing the synthesis of ginsenosides	[129]
	Adventitious root	Multi-branched root induced	17.92 mg/g ginsenosides	[130]
	Adventitious root	Jasmonate treatment	71.94 mg/g ginsenosides	[131]
	Transgenic cell lines	Overexpression of *FPS* and *RNAi* of *CAS*	2.46-Fold total ginsenosides	[132]
	Transgenic cell lines	Overexpression of *PnFPS*	2.66 times Rh1, 1.76 times Rg1, 4.35 times Re, and 2.90 times Rd	[133]
	Transgenic cell lines	Overexpression of *β-AS*	Chikusetsusaponin IV and chikusetsusaponin IVa were obtained	[134]
	Transgenic cell lines	Overexpression of *PnbHLH1*	2.27 Times total saponin contents	[103]
	Transgenic cell lines	Overexpression of *PnERF1*	Total saponins increased ~2-fold from 40 to 80 mg/g	[106]
	Transgenic cell lines	Overexpression of *PnbHLH*	Total saponins increased ~2.6-fold	[104]
Yeast	*S. cerevisiae*	Co-expression of *HMGR*, *DDS* and *β-AS*, *OAS*, *PPDS*, *PPTS* and cytochrome *P450*	17.2 mg/l PPD, 15.9 mg/l PPT, and 21.4 mg/l oleanolic acid	[135]
	*S. cerevisiae*	PPDSeATR1 protein fusion and ROS tolerance enhancement	4.25 g/l PPD	[136]
	*S. cerevisiae*	Overexpression of *UGTPg45*	529.0 mg/l PPD	[137]
	*S. cerevisiae*	Overexpression of *PgUGT74AE2* and *UGTPg1*	2.4 g/l 3b-O-Glc-DM and 5.6 g/l 20S-O-Glc-DM	[138]
	*S. cerevisiae*	Overexpression of *UGTPg1*	1.4 mg/l compound K	[139]
	*S. cerevisiae*	Overexpression of *Pn3-32-i5*	>1 g/l compound K	[140]
	*S. cerevisiae*	Overexpression of *tHMG1*	9.05 mg/l 3β,12β-Di-O-Glc-PPD	[141]
	*S. cerevisiae*	Overexpression of *PgUGT71A53* and *PgUGT71A54*	1.95 g/L Rg1	[142]
Bacteria and fungi	*Dictyoglomus turgidum* and *Pyrococcus furiosus*	Conversion of main ginsenosides to minor ginsenosides	210 mg/l/h aglycon protopanaxatriol	[143]
	*Cladosporium xylophilum*	Transformation of main ginsenosides to minor ginsenosides	0.99 mg/ml F2, 0.67 mg/ml Rd2, 0.24 mg/ml Fd, and 0.24 mg/ml Fe	[144]
	β-Glucosidase (Bgp1)	Ginsenosides Re and Rg1 to ginsenosides Rg2 and Rh1	0.83 mg/ml Rg2 and 0.6 mg/ml Rh1	[145]
	*Enterobacter chengduensis*; *Trichoderma koningii*; *Penicillium chermesinum*	Biotransformation of main ginsenosides to minor ginsenosides	Rg1 to F1 at a rate of 13.24%; Rb1 to Rd (40.00%); Rb1 to Rg3 (32.31%); Rb1 to Rd (74.24%)	[146]
	*Coniochaeta* sp.	Conversion of ginsenoside Rb1 to C-K	Conversion rate of 11. 62%	[147]
	*Pestalotiopsis biciliata*	Conversion of ginsenoside Rb1 into rare ginsenoside F2, C-K, and ginsenoside Rd	Conversion rates of 53, 11 and 10%, respectively	[148]
	*Bifidobacterium lactis* Bi-07	Transformation of ginsenosides Rd to F2	Conversion rate of 25%	[149]
	*Aspergillus niger*	Ginsenoside Rb1 to generate Rg3	Conversion rate of 48.5%	[150]

## Biotechnological methods for saponin production

*Panax notoginseng* has a slow growth rate, typically requiring 3–4 years from seed germination to root harvest in field cultivation. Additionally, challenges such as plant pathogens and pests must be managed during the cultivation process. Metabolic engineering offers a viable alternative for enhancing the production of natural products. Consequently, biotechnological approaches, including tissue culture, adventitious roots, transgenic plants, and microbial cell factories, are recommended to boost ginsenoside production (Table 2).

### Tissue culture

Suspension culture is an effective method for producing ginsenosides in *P. notoginseng*. The components of the medium, such as sugar types, significantly impact the ginsenoside concentration in cultured cells. Specifically, 2-hydroxyethyl jasmonate (HEJA) enhances the activity of protopanaxadiol 6-hydroxylase, thereby increasing the contents of Rb1 and Rg1 in cell cultures [125]. HEJA is more effective than MeJA in stimulating ginsenoside biosynthesis and altering its composition. Treatment with HEJA results in a 60% increase in total ginsenoside content and a 30% increase in the Rb/Rg ratio [126]. Additionally, adding 1 mM phenobarbital to the cultures boosts the levels of PPT-type ginsenosides (Rg1 + Re) [127]. Introducing 200 μM MeJA to the cultures can raise the levels of both PPD-type and PPT-type ginsenosides 9- and 2-fold, respectively [128]. Plant hormones such as HEJA, endogenous jasmonic acid (JA), and MeJA can induce the upregulation of squalene epoxidase (*SE*) and the suppression of cycloartenol synthase (*CAS*), promoting ginsenoside synthesis in *P. notoginseng* cells [129].

Hairy root and adventitious root cultures have been effectively used to produce stable biomass and high production of ginsenosides in *Panax* plants for over 15 years [151]. Research has revealed that an adventitious root line derived from wild-type roots of *P. notoginseng* has a high total ginsenoside yield of 17.92 mg/g [130]. In *P. notoginseng* adventitious roots, the highest total ginsenoside content reaches 71.94 mg/g after treatment with 5 mg/l JA, marking an 8.45-fold increase compared with the control [131]. Both JA and methyl dihydrojasmonate significantly boost Rd and Rg contents in these roots [131].

### Transgenic plants

Despite considerable efforts to produce ginsenosides through tissue and cell culture, the yield remains relatively low. Employing genetic engineering to manipulate gene expression has proven to be an effective strategy for enhancing ginsenoside content. Transgenic *P. notoginseng* cell lines show higher expression of farnesyl pyrophosphate synthase (*FPS*) and lower expression of cycloartenol synthase (*CAS*) compared with wild-type cells, resulting in increased total ginsenoside production and decreased phytosterol levels [132]. In transgenic *FPS*-positive *P. notoginseng* cell lines, both relative *PnFPS* expression and ginsenoside contents are significantly higher, with increases of 2.66 times for Rh1, 1.76 times for Rg1, 4.35 times for Re, and 2.90 times for Rd, than in non-transgenic controls [133]. Moreover, enhancing β-amyrin synthase (*β-AS*) expression in transgenic *P. notoginseng* cells elevates the expression of key enzymes involved in ginsenoside biosynthesis and increases the levels of specific ginsenosides such as chikusetsusaponin IV and IVa [134]. Additionally, when three enzyme genes (*PnDDS*, *CYP12H*, and *UGTPn3*) were introduced into tobacco the three exogenous genes were expressed in the roots, stems, and leaves of the transgenic plants, and thus ginsenoside Rh2 and its precursors were successfully synthesized [152].

TFs such as PnbHLH1 and PnERF1 are known to enhance ginsenoside biosynthesis and accumulation. In *P. notoginseng* cell lines engineered to express *PnbHLH1*, the total ginsenoside content is more than double that of the control, specifically 2.27 times greater [103]. Similarly, in cell lines with *PnERF1* transgenic modifications, total ginsenoside content approximately doubles from 40 in control lines to 80 mg/g [106]. Additionally, both RNAi targeting *PnCAS* and overexpression of *PnbHLH* in *P. notoginseng* cell lines result in increased levels of total and individual ginsenosides (Rd, Rb1, Re, Rg1, and R1) compared with wild-type and *PnCAS* RNAi cells [104].

### Microbial cell factories

#### Yeast cell factories

Yeast cell factories, particularly those based on synthetic biology and heterogeneous expression systems, are proving effective for synthesizing various natural products [153]. For instance, co-expression of *HMGR*, *DS*, *β-AS*, *OAS*, *PPDS*, *PPTS*, and cytochrome P450 genes in *Saccharomyces cerevisiae* can enable the simultaneous production of PPD (17.2 mg/l), PPT (15.9 mg/l), and oleanolic acid (21.4 mg/l) [135]. Enhanced fed-batch fermentation techniques incorporating PPDSeATR1 protein fusion and ROS tolerance in *S. cerevisiae* boost PPD levels up to 4.25 g/l [136]. A newly developed yeast chassis can achieve 529.0 mg/l PPD in shake flasks and 11.02 g/l in 10-l fed-batch fermentations [137]. Additionally, 3b-O-Glc-DM and 20S-O-Glc-DM ginsenosides can reach concentrations of 2.4 and 5.6 g/l, respectively, via fed-batch fermentation [138]. Engineering yeast strains with *UGTPg1* led to the production of ginsenoside compound K at a concentration of 1.4 mg/l [139]. A novel UDP-xylose-dependent glycosyltransferase enzyme, Pn3-32-i5, identified for R1 biosynthesis, allows a yeast cell factory to yield over 1 g/l of ginsenoside compound K [140]. Furthermore, overexpression of *tHMG1* in yeast increases the production of 3β,12β-Di-O-Glc-PPD from 6.17 to 9.05 mg/l [141]. Finally, Rg1-producing yeast strains, incorporating *PgUGT71A53* and *PgUGT71A54*, can produce Rg1 at 1.95 g/l [142].

#### Bacterial and fungal cell factories

Rare ginsenosides, which are more readily absorbed into the bloodstream and act as active substances, have garnered increasing attention. Microbial fermentation is utilized for producing these rare ginsenosides through the hydrolysis of substrate ginsenosides by microbial enzymes. For instance, enzymes from *Dictyoglomus turgidum* and *Pyrococcus furiosus* achieve a complete conversion of PPT-type ginsenosides (such as R1, R2, Re, Rg1, Rg2, Rh1, Rf, F1, F3, and F5) into 210 mg/l of aglycon PPT [143]. Additionally, *Cladosporium xylophilum* converts main ginsenosides Rb1, Rb2, Rb3, Rc, and notoginsenoside Fa into minor ginsenosides F2 (0.99 mg/ml), Rd2 (0.67 mg/ml), Fd (0.24 mg/ml), and Fe (0.24 mg/ml) [144]. Recombinant β-glucosidase has been used to transform Re and Rg1 into Rg2 (0.83 mg/ml) and Rh1 (0.6 mg/ml), respectively [145]. *Enterobacter chengduensis* converts Rg1 into F1 with a conversion rate of 13.24%; *Trichoderma koningii* converts Rb1 into Rd (40.00%) and Rg3 (32.31%); and *Penicillium chermesinum* transforms Rb1 into Rd at a high rate of 74.24% [146]. The endophyte *Coniochaeta* sp. can convert Rb1 into the rare ginsenoside CK with an 11.62% conversion rate [147]. *Pestalotiopsis biciliata* converts Rb1 into rare ginsenosides F2, CK, and Rd, with conversion rates of 53, 11, and 10% respectively [148]. *Bifidobacterium lactis* transforms Rb1, Rc, and Rb2 into Rd, and further into the rare ginsenoside F2, achieving a conversion rate of 25% [149]. β-Glucosidase from *Aspergillus niger* hydrolyzes Rb1 to produce Rg3 at a conversion rate of 48.5% [150].

## Conclusions and future perspectives

*Panax notoginseng* and its secondary metabolites are invaluable for human therapy and healthcare, leading to an increased use of this plant as a primary ingredient in numerous products. Consequently, demand for *P. notoginseng* continues to rise. However, limited varieties, challenges with continuous cropping, and other issues pose significant obstacles to the sustainable development of the *P. notoginseng* industry. Cultivating new and high-quality varieties is crucial for the ongoing sustainability and growth of this sector.

### Modern omics technology accelerates breeding of *P. notoginseng*

Currently, the affordability and accuracy of high-throughput sequencing have enhanced the feasibility of accessing gene resources. Utilizing high-throughput sequencing technologies and bioinformatics to deeply explore genomic, transcriptomic, and proteomic data of plants aids in the discovery of novel genes. Advanced assays such as quantitative mass spectrometry-based, fluorescence-based, and other high-throughput methods enable rapid detection of enzyme activity and efficient screening of genes. Modern omics technologies are pivotal in accelerating the breeding of new *P. notoginseng* varieties and providing valuable germplasms for the sustainable development of the *P. notoginseng* industry.

### Transcriptional regulatory networks reveal synthesis mechanisms

TFs play crucial roles in regulating both biotic and abiotic stresses during the growth of *P. notoginseng* plants, with ginsenoside accumulation closely tied to their regulatory functions and environmental adaptability. Efforts to construct a co-expression network of TFs and gene expression patterns in *P. notoginseng* have been made; however, the intricate regulatory mechanisms of these TFs require further exploration. Discovering novel genes and TFs will significantly enhance molecular plant breeding. Many TFs involved in ginsenoside production also control multiple genes simultaneously, highlighting the need for a comprehensive understanding of the molecular mechanisms that influence transcriptional regulation of ginsenosides to develop innovative breeding strategies.

### Synthetic biology increases ginsenoside production

Given that the cultivation of *P. notoginseng* is both time-consuming and labor-intensive, the development of bioengineering approaches, such as tissue culture, adventitious roots, transgenic plants, and microbial cell factories, has been pursued to enhance ginsenoside production. Thoroughly understanding the biosynthesis and regulatory mechanisms of ginsenosides will greatly benefit their biotechnological production. These advancements provide an affordable and efficient industrial platform for producing ginsenosides. Identifying new ginsenoside synthesis is expected to pave the way for novel methods that facilitate efficient and large-scale production of ginsenosides.

## Supplementary Material

Web_Material_uhae170

## Data Availability

The accession number of the RNA sequencing data is PRJNA488357.
