# Supplementary material for: Panax notoginseng: panoramagram of phytochemical and pharmacological properties, biosynthesis, and regulation and production of ginsenosides
Source: Hortic Res. 2024 Jul 2;11(8):uhae170. doi: 10.1093/hr/uhae170 (PMC11317898; doi:10.1093/hr/uhae170)
Supplement: Web_Material_uhae170 [file web_material_uhae170.zip › Supplemental file 1.doc]

**Supplemental list**

**Figure S1** Chemical structures of dencichine (β-N-oxalo-L-α-β-diaminopropionic acid)

**Table S1** The chemical constituents isolated from *Panax notoginseng*

**Figure S1** Chemical structures of dencichine (β-N-oxalo-L-α-β-diaminopropionic acid)

**Table S1** The chemical constituents isolated from *Panax notoginseng*

| **No.** | **Chemical components** | **Part of the plant** | **Representative references** |
| --- | --- | --- | --- |
|  | **Saponins** |  |  |
| 1 | Ginsenoside Ra3 | Ro; Ro&Rh | [1,2] |
| 2 | Ginsenoside Rb1 | Ro; HRo; Ro&Rh; Rh; L; St&L; Fr; Fp; Fb; Se; | [1-4] |
| 3 | Ginsenoside Rb2 | Ro; Ro&Rh; Rh; L; Fb; Fp | [2] |
| 4 | Ginsenoside Rb3 | Ro; St&L; L; Fb; Fr; Fp; Se | [4-6] |
| 5 | Ginsenoside Rc | St & L; L; Fb; Fp; Se | [3,6] |
| 6 | Ginsenoside Rd | Ro; HRo; Ro&Rh; Rh; L; St& L; Fb; Fr; Fp; Se | [1-4,7] |
| 7 | Ginsgenoside Rg3 | Ro; HRo; FRo; Ro& Rh; Rh; L; St & L | [2,6] |
| 8 | Ginsenoside compound K | L | [5] |
| 9 | Ginsenoside F2 | Ro; Ro & Rh; Rh; L; St & L; Fb; Fp | [2,6,7] |
| 10 | Ginsenoside Mc | L | [3] |
| 11 | Ginsenoside Rh2 | L; St & L | [3,6] |
| 12 | Malonyl-ginsenoside Rb1 | Ro | [8] |
| 13 | Notoginsenoside D | Ro & Rh; Ro; Fb | [1,2] |
| 14 | Notoginsenoside Fa | Ro; Ro & Rh; Rh; L; St & L; Fb; Fp; Se | [1-3,9] |
| 15 | Notoginsenoside Fc | St & L; L; Fb; Fr; Fp; Se | [3,6,9] |
| 16 | Notoginsenoside Fe | L; Fb; Fp | [3] |
| 17 | Notoginsenoside FP2 | L; Fb; Fp | [5] |
| 18 | Notoginsenoside O | Fb | [10] |
| 19 | Notoginsenoside P | Fb | [10] |
| 20 | Notoginsenoside Q | Fb | [11] |
| 21 | Notoginsenoside R4 | Ro; HRo; Rh; Ro & Rh | [2] |
| 22 | Notoginsenoside S | Rh; Fb | [12] |
| 23 | Notoginsenoside ST-4 | Ro | [13] |
| 24 | Notoginsenoside T | Ro; Rh; Fb | [8] |
| 25 | Gypenoside IX | L; St & L; Fb; Fp; Se | [3,6] |
| 26 | Gypenoside XIII | L; St & L; Fp | [14,15] |
| 27 | Gypenoside XVII | Ro; HRo; Ro & Rh; Rh; L; St & L; Fb; Fp | [2,14,16] |
| 28 | Notoginsenoside L | Ro | [17] |
| 29 | Quinquenoside R1 | Ro | [5,8] |
| 30 | Vina-ginsenoside R7 | L; Fp | [3,15] |
| 31 | Notoginsenoside K | Ro | [5] |
| 32 | Ginsenoside Rs3 | Ro; L | [6,18] |
| 33 | Compound P6 | L | [19] |
| 34 | Ginsenoside Ra1 | L | [19] |
| 35 | Gypenoside XV | Fp | [20] |
| 36 | Notoginsenoside FZ | L | [3] |
| 37 | 20(S)-protopanaxadiol | Fr; L | [3,4] |
| 38 | Chikusetsusaponin L5 | Ro; Fp | [8,15] |
| 39 | Ginsenoside F1 | Ro; Rh; L; Fp | [1,3] |
| 40 | Ginsenoside Re | Ro; HRo; Ro & Rh; Rh; L; St & L; Fb; Fp; Fr | [1,2,4,9] |
| 41 | Ginsenoside Rf | Ro; Rh | [12,21] |
| 42 | Ginsenoside Rg1 | Ro; HRo; Ro&Rh; Rh; L; St & L; Fb; Fp | [1,2,7] |
| 43 | 20(S)-Ginsenoside Rg2 | Ro; Ro & Rh; Rh; Fr | [1,2,7] |
| 44 | Ginsenoside Rh1 | Ro; HRo; FRo; Ro & Rh; Rh; L; Fb | [1-3,7] |
| 45 | 20-O-glucoginsenoside Rf | Ro; Rh | [12] |
| 46 | Koryoginsenoside R1 | Ro; Rh | [12,21] |
| 47 | Notoginsenoside FP1 | Fp | [15] |
| 48 | Notoginsenoside M | Ro | [15] |
| 49 | Notoginsenoside N | Ro | [21] |
| 50 | Notoginsenoside R1 | Ro; HRo; Ro & Rh; Rh; L; St & L; Fb; Fp | [1,2,5,7] |
| 51 | Notoginsenoside R2 | Ro; Ro & Rh; Rh; FRo | [1,2] |
| 52 | Notoginsenoside R3 | Ro | [22] |
| 53 | Notoginsenoside R6 | Ro | [22] |
| 54 | Notoginsenoside RW-1 | Rh | [5] |
| 55 | Notoginsenoside U | Ro | [23] |
| 56 | Yesanchinoside D | Ro | [24] |
| 57 | Pseudoginsenoside RT3 | Ro | [6,21] |
| 58 | 20(*S*)-6-O-[β-D-xylopyranosyl-(1→2)-β-D-xylopyranosyl]dammar-24-ene-3β,6α,12β,20-tetrol | Ro | [1] |
| 59 | 20(*S*)-protopanaxtRool | Ro; L | [6,24] |
| 60 | 20(*S*)-20-O-[β-D-xylopyranosyl-(1→6-β-D-glucopyranosyl-(1→6)-β-D-glucopyranosyl]dammar-24-ene-3β,6α,1  2β,20-tetrol | Ro | [1] |
| 61 | 6’-O-Acetyl ginsenoside Rh1 | Ro; Ro & Rh | [1,2] |
| 62 | 20(*S*)-protopanaxatRool-20-O-β-D-glucopyranosyl-(1→6)-β-D-glucopyranoside | Ro | [1] |
| 63 | 20(*S*)-sanchirhinosides A1 | Ro | [25] |
| 64 | 20(*S*)-sanchirhinosides A2 | Ro | [25] |
| 65 | 20(*S*)-sanchirhinosides A3 | Ro | [25] |
| 66 | 20(*S*)-sanchirhinosides A4 | Ro | [25] |
| 67 | 20(*S*)-sanchirhinosides A5 | Ro | [25] |
| 68 | 20(*S*)-sanchirhinosides A6 | Ro | [25] |
| 69 | 6-O-(β-D-glucopyranosyl)-20-O-(β-D-xylopyranosyl)-3β,6α,12β,20(*S*)-tetrahydroxydammar-24-ene | Ro | [1,6] |
| 70 | Notoginsenoside A | Ro | [20] |
| 71 | Notoginsenoside B | Ro | [20] |
| 72 | Notoginsenoside C | Ro | [20] |
| 73 | Notoginsenoside E | Ro; Rh | [5,26] |
| 74 | Ginsenoside II | Rh | [26] |
| 75 | Floranotoginsenosides B | Fl | [27] |
| 76 | Gypenoside LXXI | Fl | [27] |
| 77 | Notoginsenosides SFt1 | L | [6] |
| 78 | Notoginsenosides SFt2 | L | [6] |
| 79 | Floranotoginsenosides A | Fl | [27] |
| 80 | Floranotoginsenosides C | Fl | [27] |
| 81 | Floranotoginsenosides D | Fl | [27] |
| 82 | Gypenoside LXIX | Fl | [27] |
| 83 | 3β,12,20(*S*)-tRohydroxy-25-hydroperoxydammar-23-ene-3-O-[β-D–  glucopyranosyl(1→2)-β-D-glucopyranosyl]-20-O-[β-D-xylopyranosyl(1→6)]-β-D-glucopyranoside | Fl | [27] |
| 84 | Floraginsenoside O | Fl | [27] |
| 85 | Ginsenoside V | Ro | [16] |
| 86 | Notoginsenoside H | Ro | [5]) |
| 87 | Notoginsenoside J | Ro | [5] |
| 88 | Notoginsenoside RW-2 | Rh | [12,28] |
| 89 | Vinaginsenoside R15 | Ro | [6] |
| 90 | Notopanaxoside A | Ro | [20,29] |
| 91 | Vinaginsenoside R22 | Ro | [21] |
| 92 | Notoginsenoside R8 | Ro | [30] |
| 93 | Ginsenoside Rg5 | Ro; Ro & Rh; L | [2,6] |
| 94 | Ginsenoside Rh4 | Ro; Ro & Rh; Rh; Se; FRo | [1,2] |
| 95 | Ginsenoside Rk1 | Ro; Ro & Rh; L | [2,6] |
| 96 | Ginsenoside Rk3 | Ro; Ro & Rh | [2,21] |
| 97 | Notoginsenoside R7 | Ro | [20] |
| 98 | Notoginsenoside ST-1 | Ro | [24] |
| 99 | Notoginsenoside ST-2 | Ro | [24] |
| 100 | Notoginsenoside ST-3 | Ro | [24] |
| 101 | Notoginsenoside ST-5 | Ro | [24] |
| 102 | Notoginsenoside T5 | Rh | [12] |
| 103 | Pn-1 | L | [31] |
| 104 | Sanchinoside B1 | Ro; HRo | [24] |
| 105 | 20(*S*)-Panaxadiol | Rh; L; Fr | [3,4] |
| 106 | 3β,6α,12β-tRohydroxydammar-20(21),24-diene | Ro | [24] |
| 107 | Ginsenoside R10 | L | [6] |
| 108 | Vinaginsenoside R4 | Ro | [1] |
| 109 | Ginsenoside Rk2 | L | [6] |
| 110 | Ginsenoside Rs5 | L | [6] |
| 111 | Ginsenoside Rh3 | L | [6] |
| 112 | Ginsenoside Rs4 | L | [6] |
| 113 | Pseudoginsenoside F11 | Ro | [32] |
| 114 | 3-O-β-D-glucopyranosyl-(1→2)-β-D-glucopyranoside-12β,25-dihydroxydammar-(E)20(22)-ene | Ro | [33] |
| 115 | Ginsenoside F4 | Ro | [18] |
| 116 | Notoginsenoside Spt1 | Ro | [18] |
| 117 | 20(*S*)-panaxatriol | Rh | [20] |
| 118 | Sanchirhinoside D | Ro | [16] |
| 119 | Pseudoginsenoside Rt5 | Ro | [21] |
| 120 | 3β,12β-dihydroxydammar-(E)-20(22),24-diene-6-O-β-D-xylopyranosyl-(1→2)-β-D-glucopyranoside | Ro | [21] |
| 121 | Notoginsenoside SFt3 | L | [6] |
| 122 | Notoginsenoside SFt4 | L | [6] |
| 123 | Notoginsenoside R10 | Ro | [34] |
| 124 | 20(*R*)-Ginsenoside Rg3 | Ro; Rh; Ro & Rh; L; Fb; Fr | [1,2,9] |
| 125 | 20(*R*)-Ginsenoside Rh2 | Fr; L | [3,4] |
| 126 | Notoginsenoside Ft1 | L | [6] |
| 127 | 6″-O-acetylginsenoside Rg3 | Ro | [24] |
| 128 | 20(*R*)-protopanaxadiol | Rh; L | [3] |
| 129 | 3β,6α-20(*S*)-6,20-bis(β-Dglucopyranosyloxy)-  3-hydroxy dammar-24-en-12-one | Ro | [16] |
| 130 | Notoginsenoside I | Ro | [5] |
| 131 | Vinaginsenoside R3 | Ro | [6] |
| 132 | 5,6-didehydroginsenoside Rd | Ro | [8] |
| 133 | Notoginsenoside G | Ro | [1,5] |
| 134 | 5,6-didehydroginsenoside Rb1 | Ro | [8] |
| 135 | 20(*R*)-protopanaxtriol | Ro; L; Rh | [3,6] |
| 136 | 20(*R*)- Ginsenoside Rg1 | F1 | [4] |
| 137 | 20(*R*)-Ginsenoside Rh1 | Ro; Ro & Rh; L; Fr | [1-3] |
| 138 | Ginsenoside U | Ro | [21] |
| 139 | 25-hydroxy-20(*R*)-ginsenoside Rh1 | Ro | [24] |
| 140 | Notoginsenoside R9 | Ro | [30] |
| 141 | Notoginsenoside-LX | L | [3] |
| 142 | Notoginsenoside-LY | L | [3] |
| 143 | Sanchirhinoside B | Ro | [25] |
| 144 | 6-O-β-D-glucopyranosyl-20-O-β-D-glucopyranosyl-20(*S*)-protopanaxadiol-3-one | Ro | [35] |
| 145 | Lupeol | Se; Fr | [36] |
| 146 | Betulin | F1 | [37] |
| 147 | 16β-hydroxy lupeol | Se; Fr | [36] |
| 148 | Lup-20-ene-3β,16β-diol-3-ferulate | Se | [36] |
| 149 | Notoginsenoside Rw-3 | Rh | [5] |
| 150 | Notoginsenoside Rw-4 | Rh | [5] |
| 151 | Notoginsenoside Rw-5 | Rh | [5] |
| 152 | Notoginsenoside RW-6 | Rh | [5] |
| 153 | Notoginsenoside Rw-7 | Rh | [5] |
| 154 | Notoginsenoside Fh-1 | L | [38] |
| 155 | Notoginsenoside Fh-2 | L | [38] |
| 156 | Notoginsenoside Fh-3 | L | [38] |
| 157 | Notoginsenoside Fh-4 | L | [38] |
| 158 | Notoginsenoside Fh-5 | L | [38] |
| 159 | Notoginsenoside Fh-6 | L | [38] |
| 160 | Notoginsenoside Fh-8 | L | [39] |
| 161 | Notoginsenoside Fh-9 | L | [39] |
| 162 | Notoginsenoside Ng-1 | L | [40] |
| 163 | Notoginsenoside Ng-2 | L | [40] |
| 164 | Notoginsenoside Ng-3 | L | [41] |
| 165 | Notoginsenoside Ng-4 | L | [41] |
| 166 | Nototroneside A | L | [42] |
| 167 | Nototroneside B | L | [42] |
| 168 | Nototroneside C | L | [42] |
| 169 | Notoginsenoside LK-1 | L | [43] |
| 170 | Notoginsenoside LK-2 | L | [43] |
| 171 | Notoginsenoside LK-3 | L | [43] |
| 172 | Notoginsenoside LK-4 | L | [43] |
| 173 | Notoginsenoside LK-5 | L | [43] |
| 174 | Notoginsenoside LK-6 | L | [43] |
| 175 | Notoginsenoside LK-7 | L | [43] |
| 176 | Notoginsenoside LK-8 | L | [43] |
| 177 | Notoginsenoside NL-A1 | L | [44] |
| 178 | Notoginsenoside NL-A2 | L | [44] |
| 179 | Notoginsenoside NL-A3 | L | [44] |
| 180 | Notoginsenoside NL-A4 | L | [44] |
| 181 | Notoginsenoside NL-B1 | L | [44] |
| 182 | Notoginsenoside NL-B2 | L | [44] |
| 183 | Notoginsenoside NL-B3 | L | [44] |
| 184 | Notoginsenoside NL-C1 | L | [44] |
| 185 | Notoginsenoside NL-C2 | L | [44] |
| 186 | Notoginsenoside NL-C3 | L | [44] |
| 187 | Notoginsenoside NL-D | L | [44] |
| 188 | Notoginsenoside NL-E1 | L | [45] |
| 189 | Notoginsenoside NL-E2 | L | [45] |
| 190 | Notoginsenoside NL-E3 | L | [45] |
| 191 | Notoginsenoside NL-E4 | L | [45] |
| 192 | Notoginsenoside NL-F1 | L | [45] |
| 193 | Notoginsenoside NL-F2 | L | [45] |
| 194 | Notoginsenoside NL-G1 | L | [45] |
| 195 | Notoginsenoside NL-G2 | L | [45] |
| 196 | Notoginsenoside NL-H1 | L | [45] |
| 197 | Notoginsenoside NL-H2 | L | [45] |
| 198 | Notoginsenoside NL-H3 | L | [45] |
| 199 | Notoginsenoside NL-I | L | [46] |
| 200 | Notoginsenoside NL-J | L | [46] |
| 201 | Gypenoside PN02 | Fp; L | [47] |
| 202 | Gypenoside PN03 | Fp; L | [47] |
| 203 | Notoginsenoside SY1 | Ro | [48] |
| 204 | Notoginsenoside SY2 | Ro | [48] |
| 205 | Notoginsenoside SY3 | St; L | [49] |
| 206 | Notoginsenoside SY4 | St; L | [49] |
| 207 | Notoginsenoside SY5 | St; L | [49] |
| 208 | Notoginsenoside Ab1 | St; L | [49] |
| 209 | Notoginsenoside Ab2 | PNS | [50] |
| 210 | Notoginsenoside Ab3 | PNS | [50] |
| 211 | Notoginsenoside SL1 | L | [51] |
| 212 | Notoginsenoside SL2 | L | [51] |
| 213 | Notoginsenoside SL3 | L | [51] |
| 214 | Notoginsenoside SL4 | L | [51] |
| 215 | Notoginsenoside SL5 | L | [51] |
| 216 | Notoginsenoside SL6 | L | [51] |
| 217 | Notoginsenoside SL7 | L | [51] |
| 218 | Notoginsenoside SL8 | L | [51] |
|  | **Amino acids** |  |  |
| 1 | Dencichine | Ro, L, Fl, Rh, | [52] |
| 2 | Arginine | Ro, HRo | [53,54] |
| 3 | Aspartic acid | Ro, HRo, Fl | [53,54] |
| 4 | Glutamic acid | Ro, HRo, Fl | [53,54] |
| 5 | Leucine | Ro, HRo, Fl | [53,54] |
| 6 | Lysine, | Ro, HRo, Fl | [53,54] |
| 7 | Phenylalanine | Ro, HRo, Fl | [53,54] |
| 8 | Alanine, | Ro, HRo, Fl | [53,54] |
| 9 | Valine | Ro, HRo, Fl | [53,54] |
| 10 | Proline | Ro, HRo, Fl | [53,54] |
| 11 | Threonine | Ro, HRo, Fl | [53,54] |
| 12 | Isoleucine acid | Ro, HRo, Fl | [53,54] |
| 13 | Glycine | Ro, HRo, Fl | [53,54] |
| 14 | Serinethe | Ro, HRo, Fl | [53,54] |
|  | **Polysaccharides** |  |  |
| 1 | Sanchinan-A | Fro | [55] |
| 2 | PF3111 | Ro | [56] |
| 3 | PF3112 | Ro | [56] |
| 4 | PBGA11 | Ro | [56] |
| 5 | PBGA12 | Ro | [56] |
| 6 | AIR | Fro | [57] |
| 7 | Fr1MKOH | Fro | [58] |
| 8 | 1MD3-G2 | Fro | [58] |
| 9 | Arabinoglucogalactan  (1) | Ro | [58] |
| 10 | MRP | Fro | [59] |
| 11 | BRP | Fro | [59] |
| 12 | FRP | Fro | [59] |
| 13 | RN1 | Fl | [60] |
| 14 | N1 | Ro | [61] |
| 15 | N2 | Ro | [61] |
| 16 | N3 | Ro | [61] |
| 17 | N4 | Ro | [61] |
| 18 | N5 | Ro | [61] |
| 19 | MRP5A | Ro | [62] |
| 20 | MRP5 | Ro | [62] |
| 21 | PNPS-0.5M | RPN | [63] |
| 22 | PNPS-0.3 | RPN | [64] |
| 23 | PNPN | Ro | [65] |
| 24 | PNPA-1A | Ro | [65] |
| 25 | PNPA-2A | Ro | [65] |
| 26 | PNPA-3A | Ro | [65] |
| 27 | PNPA-1B | Ro | [65] |
| 28 | PNPA-2B | Ro | [65] |
| 29 | PNPA-3B | Ro | [65] |
| 30 | PPN | Ro | [66] |
| 31 | NPPN | RPN | [67] |
| 32 | APPN I | RPN | [67] |
| 33 | APPN II | RPN | [67] |
| 34 | APPN III | RPN | [67] |
| 35 | APPN IV | RPN | [67] |
| 36 | FPNP | Ro | [68] |
| 37 | APPN I | RPN | [69] |
| 38 | APPN II-A | RPN | [69] |
| 39 | APPN II-B | RPN | [69] |
| 40 | APPN III-A | RPN | [69] |
| 41 | APPN III-B | RPN | [69] |
|  | **Flavonoids** |  |  |
| 1 | Liquiritigenin | L; St & L | [14,70] |
| 2 | Liquiritin apioside | L; St & L | [14,70] |
| 3 | Quercetin | HRo ; St & L | [7,71] |
| 4 | Quercetin-3-O-Sophoroside | L | [7] |
| 5 | Quercetin3-O-(2″-β-D-glucopyranosyl-β-D-galactopyranoside | Fb; St & L | [7,72] |
| 6 | Quercetin3-O-β-D-xylopyranosyl-β-D-galactopyranoside | Ro | [73] |
| 7 | Kaempferol | St & L; Se | [74] |
| 8 | Kaempferol-7-O-α-L-rhamnoside | Se & L | [71] |
| 9 | Kaempferol-3-O-β-D-galactoside | Se & L | [71] |
| 10 | Kaempferol-3-O-α-L-rhamnoside | Fb | [7] |
| 11 | Kaempferol3-O-(2”-β-D-glucopyranosyl)-β-D-galactopyranoside | Fb; Se & L; Fp | [7,75] |
| 12 | Quercetin3-O-β-D-glucopyranosyl-(1→2)-β-D-galactopyranoside | Fp | [15] |
| 13 | Kaempferol-3-O-(2″,3″-di-E-p-coumaroyl)-α-L-rhamnoside | Fb | [7,76] |

Notes: Ro, Root; Rh, Rhizome; HRo, Hair Ro; L, Leaf; St, Stem; Se, Seed; Fr, Fruit; Fl, Flower; FP, Flower pedicel; Fb, Flower bud; Fro, Fermented Ro; PNS, *Panax notoginseng* saponins; RPN, Residue of *Panax notoginseng*

**References**

[1]Xie JZ, Miao JH, Yao XS. New dammarane type saponins from the roots of *Panax notoginseng*. Helv Chim Acta. 2014;97(1):102–111.

[2]Yu HS, Zhang LJ, Song XB, et al. Chemical constituents from processed rhizomes of *Panax notoginseng*. China J Chin Mat Med 2013;38:3910–3917.

[3]Li DW, Cao JQ, Bi XL, et al. New dammarane-type triterpenoids from the leaves of *Panax notoginseng* and their protein tyrosine phosphatase 1B inhibitory activity. J Ginseng Res. 2014;38:28–33.

[4]Shi SM, Li W, Cao JQ, et al. The chemical constituents study of the fruit of *Panax notoginseng*. Chin Trad Herb Drugs. 2010;42:1249–1251.

[5]Guo JJ, Li LL, Zhu HC, et al. The progress on the chemical constituents and immunoregulation effect of *Panax notoginseng*. J Tianjin Univer Trad Chin Med. 2014;33:119–124.

[6]Liu Q, Lv JL, Wang D, et al. Dammarane type saponins from steamed leaves of *Panax notoginseng*. Natur Prod Bioprosp. 2011;1:124–128.

[7] Xia PG, Zhang SC, Liang ZS, et al. Research history and overview of chemical constituents of *Panax notoginseng.* Chin Trad Herb Drugs. 2014;45:2564–2570.

[8]Wan JB, Zhang QW, Hong SJ, et al. 5,6-Didehydroginsenosides from the Roots of *Panax notoginseng*. Molecules. 2010;15:8169–8176.

[9]Zhang YL, Zuo DD. The modern research progress on the flower of *Panax notoginseng.* Inform Trad Chin Med. 2011;28:116–118.

[10]Yoshikawa M, Morikawa T, Kashima Y, et al. Structures of new dammarane-type triterpene saponins from the flower buds of *Panax notoginseng* and hepatoprotective effects of principal ginseng saponins. J Nat Prod. 2003;66:922–927.

[11]Li H, Deng CQ, Chen BY, et al. Total saponins of *Panax notoginseng* modulate the expression of caspases and attenuate apoptosis in rats following focal cerebral ischemia-reperfusion. J Ethnopharmacol. 2009;121:412–418.

[12]Cui XM, Jiang ZY, Zeng J, et al. Two new dammarane triterpene glycosides from the rhizomes of *Panax notoginseng*. J Asian Nat Prod Res. 2008;10:845–849.

[13]Pei Y, Du Q, Liao PY, et al. Notoginsenoside ST-4 inhibits virus penetration of herpes simplex virus in *vitro*. J Asian Nat Prod Res. 2011;13:498–504.

[14]Huang, F., Xiang, F.J., Wu, X.J., et al. The progress of saponins and its monomer separation and extraction in the leaves of *Panax notoginseng*. J Chin Med Mat. 2009;32:999–1005.

[15]Wang XY, Wang D, Ma XX, et al. Two new dammarane–type bisdesmosides from the fruit pedicels of *Panax notoginseng*. Helvetica Chimica Acta 2008;91:60–66.

[16]Kaunda JS, Wang T, Liu LL, et al. Eight darmarane-type saponins isolated from the roots of *Panax notoginseng*. Acta Pharm Sin B. 2013;3:381–384.

[17]Massayuki Y, Toshio M, Kenichi Y. Bioactive saponins and glycosides XIX *notoginseng* (3): immunological adjuvant activity of notoginsenosides and related saponins: structures of notoginsenosides-L,-M, and -N from the roots of *Panax notoginseng* (Burk.) F. H. Chen. Chem Pharm Bull. 2001;49:1452–1456.

[18]Zhang YC, Liu CM, Qi YJ, et al. Application of accelerated solvent extraction coupled with counter-current chromatography to extraction and online isolation of saponins with a broad range of polarity from *Panax notoginseng.* Sep Purif Technol. 2013;106:82–89.

[19]Guo J. Analysis of the chemical constituents on the rhizome of *Panax notoginseng*. Guide China Med. 2013;11:46–47.

[20]Zhou JM, Cui XM, Zeng J, et al. The research progress of the monomer saponins in different part of *Panax notoginseng* and its usage. J Chin Med Mater. 2007;30:1615–1618.

[21]Han LF, Kaunda JS, Liu LL, et al. Saponins from roots of *Panax notoginseng.* Chin Herb Med. 2014;6:159–163.

[22]Matsuura H, Kasai R, Tanaka O, et al. Further studies on dammarane-saponins of sanchi-ginseng. Chem Pharm Bull. 1983;31:2281–2287.

[23]Sun HX, Yang ZG, Ye YP. Structure and biological activity of protopanaxatriol-type saponins from the roots of *Panax notoginseng*. Int Immunopharmacol. 2006;6:14–25.

[24]Liao PY, Wang D, Zhang YJ, et al. Dammarane-type glycosides from steamed *notoginseng*. J Agr Food Chem. 2008;56:1751–1756.

[25]Zhang Y, Han LF, Sakah KJ, et al. Bioactive protopanaxatriol type saponins isolated from the roots of *Panax notoginseng* (Burk.) F. H. Chen. Molecules. 2013;18:10352–10366.

[26]Song JP, Zeng J, Cui XM, et al. Studies on chemical constituents from rhizomes of *Panax notoginseng* (II). J Yunnan Univer. 2007;29:287– 290.

[27]Wang JR, Yamasaki Y, Tanaka T, et al. Dammarane-type triterpene saponins from the flowers of *Panax notoginseng*. Molecules. 2009;14:2087–2094.

[28]Zhou JM, Cui XM, Zeng J, et al. Studies on minor chemical constituents from rhizomes of *Panax notoginseng*. Chin Trad Patent Med. 2008;30:1509–1511.

[29]Komakine N, Okasaka M, Takaishi Y, et al. New dammarane-type saponin from roots of *Panax notoginseng*. J Nat Med. 2006;60:135–137.

[30]Zhao P, Liu YQ, Yang CR. Minor dammarane saponins from *Panax notoginseng*. Phytochemistry. 1996;41:1419–1422.

[31]Mao Q, Yang J, Cui XM, et al. Target separation of a new anti-tumor saponin and metabolic profiling of leaves of *Panax notoginseng* by liquid chromatography with eletrospray ionization quadrupole time-of-flight mass spectrometry. J Pharmaceut Biomed. 2012;59:67–77.

[32]Wang X, Wang C, Wang J, et al. Pseudoginsenoside-F11 (PF11) exerts anti-neuroinflammatory effects on LPS-activated microglial cells by inhibiting TLR4-mediated TAK1/ IKK/NF-kB, MAPKs and Akt signaling pathways. Neuropharmacology. 2014;79:642–656.

[33]Liu LM, Zhang XQ, Wang H, et al. Minor saponins constituents from *Panax notoginseng* tap root. J China Pharm Univers. 2011;42:115–118.

[34]Li HZ, Teng RW, Yang CR. A novel hexanordammarane glycoside from the roots of *Panax notoginseng*. Chinese ChemLett. 2001;1:59–62.

[35]Fu HZ, Zhong RJ, Zhang DM, et al. A new protopanaxadiol-type ginsenoside from the roots of *Panax notoginseng*. J Asian Nat Prod Res. 2013;15:1139–1143.

[36]Song JP, Cui XM, Zeng J, et al. Studies on the lipophlic constituents of seeds of *Panax notoginseng*. Lishizhen Med Mater Med Res. 2010;21:565–567.

[37]Wei JX, Cao SM. A study on the saponins in fruit pedicels of *Panax notoginseng* (Burk.) F. H. Chen. China J Chin Mater Med. 1992;17:96–98,126.

[38]Yang QW, Huang Q Y, Hu Z P, et al. Potential neuroprotective treatment of stroke: targeting excitotoxicity, oxidative stress, and inflammation. Front Neurosci. 2019;13: 1036.

[39]Liu XY, M J, Li CJ, et al. New triterpenoid saponins from the leaves of *Panax notoginseng*. Acta Pharm Sin. 2017;52(9):1432.

[40] Huang JW, Chen JW, Li CJ, et al. Two new saponins from the leaves of *Panax notoginseng*. J Asian Nat Prod Res. 2018;20(4):337.

[41]Huang JW, Li CJ, Cao JZ, et al. New dammarane-type saponins from the leaves of *Panax notoginseng*. Chin Chem Lett. 2019;30(2):447.

[42]Liu XY, Li CJ, Chen FY, et al. Nototronesides A-C, three triterpene saponins with a 6/6/9 fused tricyclic tetranordammarane carbon skeleton from the leaves of *Panax notoginseng*. Org Lett. 2018;20(15):4549.

[43]Li J, Wang R F, Zhou Y, et al. Dammarane-type triterpene oligoglycosides from the leaves and stems of *Panax notoginseng* and their antiinflammatory activities. J Ginseng Res. 2019,43(3):377.

[44] Sun F, Ruan JY, Zhao W, et al. New dammarane-type triterpenoid saponins from *Panax notoginseng* leaves and their nitricoxide inhibitory activities. Molecules. 2019; 25(1):139.

[45] Ruan JY, Sun F, Zhang Y, et al. New 20(S)-protopanaxadiol type saponins from the leaves of *Panax notoginseng* and their potential anti-inflammatory activities. Steroids. 2020;162:108696.

[46]Ruan JY, Zhang JY, Zhang Y, et al. New 12,23-epoxydammarane type saponins obtained from *Panax notoginseng* leaves and their anti-inflammatory activity. Molecules. 2020;25(17):3784.

[47]Ma LJ, Cao JL, Meng FC, et al. Quantitative characterization of ginsenoside biotransformation in *Panax notoginseng* infloinflorescences and leaves by online two-dimensional liquid chromatography coupled to mass spectrometry. J Agric Food Chem. 2020;68(19):5327.

[48]Yang CK, Xiong J, Shen Y. Two new dammarane-type triterpenoids from the stems and leaves of *Panax notoginseng*. J Asian Nat Prod Res. 2021;23(4):341.

[49]Yang CK, Xiong J, Zhang L, et al. Two new dammaranetype triterpenoids from the steamed roots of *Panax notoginseng*. J Asian Nat Prod Res. 2021;23(10):961.

[50]Li Q, Yuan M R, Li XH, et al. New dammarane-type triterpenoid saponins from *Panax notoginseng* saponins. J Ginseng Res. 2020;44(5):673.

[51] Sun XJ, Deng HB, Shu TY, et al. Study on chemical constituents of *Panax notoginseng* leaves. Molecules. 2023;28:2194.

[52] Duan SF, Chen G, Yan J, et al. Optimization of extraction process and detection method of dencichine. Chin J Trop Crops. 2019;40(11):2255-2260.

[53]Li C, Zhang H, Ma H, et al. Analysis of amino acids in *Panax notoginseng*. Amino acids J. 1992;(4):46.

[54]Chen ZJ, Sun YQ, Dong TX, et al. Comparison of amino acids contents in *Panax notoginsneg* from different habitats. J Chin Med Mater. 2003;26(2): 86-88.

[55]Ohtani K, Mizutani K, Hatono S, et al. a reticuloendothelial system activating arabinogalactan from sanchi-ginseng (roots of *Panax notoginseng*). Planta Med. 1987;53:166–169

[56]Gao H, Wang F, Lien EJ, et al. Immunostimulating polysaccharides from *Panax notoginseng*. Pharm Res. 1996;13:1196–1200.

[57]Zhu Y, Pettolino F, Mau SL, et al. Immunoactive polysaccharide-rich fractions from *Panax notoginseng*. Planta Med. 2006;72:1193–1199.

[58]Wu Y, Wang D. Structural characterization and DPPH radical scavenging activity of an arabinoglucogalactan from *Panax notoginseng* root. J Nat Prod. 2008; 71:241–245.

[59]Feng S, Cheng H, Xu Z, et al. Thermal stress resistance and aging effects of *Panax notoginseng* polysaccharides on Caenorhabditis elegans. Int J Biol Macromol. 2015;81:188–194.

[60]Wang P, Zhang L, Yao J, et al. An arabinogalactan from flowers of *Panax notoginseng* inhibits angiogenesis by BMP2/Smad/Id1 signaling. Carbohydr Polym. 2015;121:328–335.

[61]Cheong KL, Wu DT, Deng Y, et al. Qualitation and quantification of specific polysaccharides from Panax species using GC-MS, saccharide mapping and HPSEC-RID-MALLS. Carbohydr Polym. 2016;153:47–54.

[62]Feng SL, Cheng HR, Xu Z, et al. Antioxidant and anti-aging activities and structural elucidation of polysaccharides from *Panax notoginseng* root. Process Biochem. 2019;78:189–199.

[63]Wang C, Zheng L, Liu S, et al. A novel acidic polysaccharide from the residue of *Panax notoginseng* and its hepatoprotective effect on alcoholic liver damage in mice. Int J Biol Macromol. 2020;149:1084–1097.

[64]Wang C, Zheng L, Liu S, et al. A novel acidic polysaccharide from the residue of *Panax notoginseng* and its hepatoprotective effect on alcoholic liver damage in mice. Int J Biol Macromol. 2020;149: 1084–1097.

[65]Chan MK, Yu Y, Wulamu S, et al. Structural analysis of water-soluble polysaccharides isolated from *Panax notoginseng*. Int J Biol Macromol. 2020;155:376–385.

[66]Xia YG, Li X, Yu LS, et al. Structural-fingerprinting of polysaccharides to discern *Panax* species by means of gas-liquid chromatography and mass spectrometry. Int J Biol Macromol. 2020;151:932–943.

[67]Liu YH, Qin HY, Zhong YY, et al. Neutral polysaccharide from *Panax notoginseng* enhanced cyclophosphamide antitumor efficacy in hepatoma H22-bearing mice. BMC Cancer 2021;21:37.

[68]You S, Shi X, Yu D, et al. Fermentation of *Panax notoginseng* root extract polysaccharides attenuates oxidative stress and promotes type I procollagen synthesis in human dermal fibroblast cells. BMC Complement Med. 2021;21:34.

# [69]Liu YH, Li S, Pu MD, et al. Structural characterization of polysaccharides isolated from *Panax notoginseng* medicinal residue and its protective effect on myelosuppression induced by cyclophosphamide. Chem Biodivers. 2022;19(1): e202100681.

[70]Li HZ, Zhang YJ, Yang CR. A further investigation on the chemical constituents from the leaves of *Panax notoginseng*. Nat Prod Res Develop. 2006;18:549–554.

[71]Zheng Y, Li XW, Gui MY, et al. Studies on flavonoids from stems and leaves of *Panax notoginseng*. Chin Pharma J. 2006;41:176–178.

[72] Zhang B, Chen XH, Bi KS. Isolation and identification of chemical constituents from flower buds of *Panax notoginseng*. J Shenyang Phama Univers. 2009;26:775–777.

[73] Choi RCY, Zhu JTT, Leung KW, et al. A flavonol glycoside, isolated from roots of *Panax notoginseng*, reduces amyloid-β-induced neurotoxicity in cultured neurons: signaling transduction and drug development for Alzheimer’s disease. J Alzheimer’s disease. 2010;19:795–811.

[74] Zhou JM, Cui XM, Zeng J, et al. Study on liposoluble components from seeds of *Panax notoginseng*. Res Prac Chin Med. 2008;22:8–10.

[75] Zhang B, Chen XH, Bi KS. Isolation and identification of chemical constituents from flower buds of *Panax notoginseng*. J Shenyang Pham Univers. 2009;26:775–777.

[76] Huang, J., Wang, H., Yang, X.F., et al. Isolation and identification of flavonoids from flower buds of *Panax notoginseng.* Nat Prod Res Develop. 2012;24:1060–1062.
